# Mutational Analysis of Sigma-1 Receptor’s Role in Synaptic Stability

**DOI:** 10.3389/fnins.2019.01012

**Published:** 2019-09-19

**Authors:** Daniel A. Ryskamp, Vladimir Zhemkov, Ilya Bezprozvanny

**Affiliations:** ^1^Department of Physiology, UT Southwestern Medical Center, Dallas, TX, United States; ^2^Laboratory of Molecular Neurodegeneration, Peter the Great Saint Petersburg State Polytechnic University, Saint Petersburg, Russia

**Keywords:** synaptic, docking-ligand fit, mutagenesis, pharmacology, structure–function relationship

## Abstract

Sigma-1 receptor (S1R) is an endoplasmic reticulum (ER) resident transmembrane protein. In our previous experiments, we demonstrated neuroprotective effects of pridopidine, an agonist of S1R, in cellular and animal models of Huntington’s disease (HD) and Alzheimer’s disease (AD). Consistent with previous observations, deletion of endogenous S1R with CRISPR/Cas9 in cultured hippocampal neurons resulted in fewer mushroom-shaped dendritic spines. Overexpression of human S1R restored mushroom spine density to control levels. In contrast, overexpression of S1R with the Δ31–50 deletion (linked to distal hereditary motor neuropathy) or the E102Q mutation (linked to amyotrophic lateral sclerosis) destabilized mushroom spines. Recently a crystal structure of S1R was determined in lipidic cubic phase. In the present study, we took an advantage of this structural information and performed docking studies with pridopidine and the S1R structural model. We generated a series of S1R point mutations based on residues predicted to be involved in direct association with pridopidine. We discovered that all ligand binding-site mutants were able to compensate for loss of endogenous S1R. However, most of these mutants were not able to support pridopidine-induced rescue of mushroom spines in presenilin-1-mutant cultures. Our mutational analysis was in agreement with *in silico* docking based on the published S1R crystal structure, with an exception of R119 residue. Our data also suggest that basal S1R activity is required for mature spine stability, whereas agonist-mediated S1R activity is required for stabilization of mushroom spines in the context of disease-causing mutations.

## Introduction

Sigma-1 receptor (S1R) pleiotropically promotes homeostasis in conditions of cellular stress when activated by a diverse assortment of exogenous drugs and endogenous ligands. It achieves this through its role as a ligand-operated chaperone, modulating the function of several client proteins and coordinating membrane lipid dynamics from its vantage point in the membrane of the endoplasmic reticulum (ER). S1R is particularly important in the brain where it regulates synaptic plasticity, calcium signaling, excitability, oxidative stress, secretion of neurotrophic factors, and neuronal viability (Bucolo et al., 2006; Martina et al., 2007; Tsai et al., 2009; Pal et al., 2012; Wang et al., 2012; Dalwadi et al., 2017; Maurice and Goguadze, 2017; Goguadze et al., 2019). Changes in its expression or sequence are associated with neurodegenerative phenotypes (Luty et al., 2010; Al-Saif et al., 2011; Kim et al., 2014; Li et al., 2015; Ullah et al., 2015; Gregianin et al., 2016; Horga et al., 2016; Watanabe et al., 2016; Wong et al., 2016) and S1R agonists [e.g., pridopidine and (+)-3-PPP] are broadly neuroprotective (Marrazzo et al., 2005; Meunier et al., 2006; Maurice and Su, 2009; Villard et al., 2009, 2011; Eddings et al., 2019; Maurice et al., 2019) and can normalize synaptic connectivity in mouse models of Huntington’s disease (HD) and Alzheimer’s disease (AD) (Ryskamp et al., 2017, 2019; Smith-Dijak et al., 2019). Mirroring synaptic deficits in these disorders, knockdown or Cas9-based deletion of S1R in primary neuron cultures prepared from neonatal mice causes loss of dendritic spines in striatal medium spiny neurons (MSNs) and loss of mature, mushroom-shaped spines in hippocampal neurons, but overexpression of human S1R (hS1R) can substitute for endogenous S1R and reinstate the synaptoprotective effects of pridopidine (Ryskamp et al., 2017, 2019).

The secondary structure of S1R was predicted by computational modeling (Brune et al., 2013, 2014). These models contain two transmembrane domains, in agreement with NMR spectroscopic analysis of S1R fragments (Ortega-Roldan et al., 2013, 2015) and with photoaffinity labeling studies of S1R (Pal et al., 2008). However, the recent crystal structure of S1R, which was determined in lipidic cubic phase (LCP), suggested an alternative model with a single transmembrane domain (Schmidt et al., 2016). Recently the same group resolved structures for agonist and antagonist bound forms of S1R (Schmidt et al., 2018). In this study, we took advantage of available structural information and performed docking studies with pridopidine and the S1R model. We validated predictions of the model in synaptic spine rescue experiments in wild-type (WT) and presenilin-mutant neurons. Our mutational analysis was generally in agreement with *in silico* docking based on the published S1R crystal structure. Our data also suggest that basal S1R activity is required for mushroom spine stability, whereas agonist-mediated S1R activity is required for stabilization of mushroom spines in the context of disease-causing mutations.

## Materials and Methods

### Animals

Experiments with WT (C57/B6J) and presenilin-1-M146V knock-in (PS1-KI) mice (Guo et al., 1999) were permitted by the Institutional Animal Care and Use Committee of the University of Texas Southwestern Medical Center at Dallas and followed the National Institutes of Health Guidelines for the Care and Use of Experimental Animals. Postnatal day 0–1 pups were used for primary neuron cultures, pooling mice from both genders.

### Western Blot

Protein lysates were either prepared from HEK293T cells or hippocampal cultures overexpressing hS1R with or without mutations. HEK293T cells were transiently transfected using polyethylenimine and lysates were prepared 48 h later. Hippocampal cultures were infected with lenti-viral particles as described below. Protein was extracted as described (Ryskamp et al., 2017). Protein lysates were analyzed by SDS–PAGE/Western blotting with mouse anti-S1R (1:200, Santa Cruz, sc-137075), mouse anti-tubulin (1:5000, DSHB, E7-c), and HRP-conjugated anti-mouse (111-035-144; Jackson ImmunoResearch) antibodies.

### *In vitro* Spine Loss Assays

Hippocampi were dissected from pups on postnatal day 0–1. Brain tissue was cut into small pieces, centrifuged, digested with papain, mechanically dissociated (with 5 mg/ml DNAse I), and plated on poly-D-lysine coated 12 mm coverslips. Cells were maintained at 37°C in a 5% CO2 incubator, feeding weekly by addition of 500 μl of Neuro Basal A (NBA), 2% B27 and 0.5 mM L-glutamine. Hippocampi from five to six pups were used to plate 24 wells of a 24-well plate. Hippocampal cultures were transfected on day *in vitro* (DIV) 7 with a TdTomato plasmid using high calcium phosphate to later visualize spine morphology. Starting on DIV18 cultures were treated with pridopidine (1 μM for 16 h prior to fixation). Cultures were fixed for 20 min in 4% formaldehyde plus 4% sucrose in phosphate buffered saline (PBS) (pH 7.4; 4°C) and rinsed with PBS. Coverslips were mounted at this point on microscope slides. *Z*-stacks were captured using a confocal microscope (Leica SP5; 63× glycerol objective N.A. 1.3). The density and shape of spines was quantified using NeuronStudio as described (Ryskamp et al., 2019).

### Lentivirus and CRISPR/Cas9 Preparation

We used a lenti-expression vector (FUGW^1^) to drive the expression of hS1R with or without mutations. Mutations in S1R were made using the Q5^®^ Site-Directed Mutagenesis Kit and resulting plasmid was sequenced to verify the codon change. For Cas9 experiments, a guideRNA sequence targeting exon 1 of S1R (GCAGCTTGCTCGACAGTATG) was cloned into the lenti-Guide-Puro plasmid^2^ (gS1R). A guideRNA sequence (GTGCGAATACGCCCACGCGAT) targeting the bacterial gene β-galactosidase (LacZ) was used as a negative control (gLacZ). The lenti-Cas9-Blast plasmid^3^ was used to express Cas9. To generate lentiviruses, plasmids were mixed with plasmids for Δ8.9 and vesicular stomatitis virus G-protein (VSVG) in 1 ml dulbecco’s modified eagle medium (DMEM) and 60 μl polyethylenimine (PEI) for 20 min at RT. Culture media was replaced with 11.5 ml of NBA and plasmids were added to transfect HEK293T cells. Media was collected 48 h later, centrifuged (2000 RPM for 5 min), filtered (0.45 μm pore size), aliquoted, flash-frozen, and stored at −80°C until use (100 μl on DIV7). Resulting lentiviruses exhibited selective neuronal tropism and an ∼90% neuronal transfection rate (Wu et al., 2016).

### Docking

For ligand docking studies of pridopidine, R(+)-3-(3-hydroxy- phenyl)-*N*-propylpiperidine (3-PPP) and (+)-pentazocine (PTZ), crystal structures of hS1R (PDB IDs: 5HK1, 5HK2) were used. Co-crystallized ligands were deleted from PDB structures. Docking was performed using AutoDockTools (Morris et al., 2009) with flexible ligands (e.g., pridopidine) and a rigid receptor. For definition of the ligand binding site, a rectangular box was defined around putative ligand-binding site (72 × 46 × 38 Å with 0.375 Å grid spacing). For each analysis, 50 individual docking runs were sampled and results were clustered. For ligand interaction analysis, the lowest energy cluster root-mean-square deviation of atomic positions (RMSD 0.5–2.0 Å) was selected. Docking results were visualized using UCSF Chimera developed by the Resource for Biocomputing, Visualization, and Informatics at the University of California, San Francisco, with support from NIH P41-GM103311 (Pettersen et al., 2004). The ligand binding site was defined to include residues within 4.5 Å of each ligand. To evaluate energetic contribution of each residue, alanine scanning mutagenesis was performed using ABS-scan software (Anand et al., 2014).

### Statistics

The Holm–Bonferroni method was used for multiple comparisons. ^∗^*p* < 0.05, ^∗∗^*p* < 0.01, ^∗∗∗^*p* < 0.001, ^∗∗∗∗^*p* < 0.0001.

## Results

### Disease Linked Mutations in S1R Destabilize Mushroom Spines in Hippocampal Neurons

**FIGURE 1:**
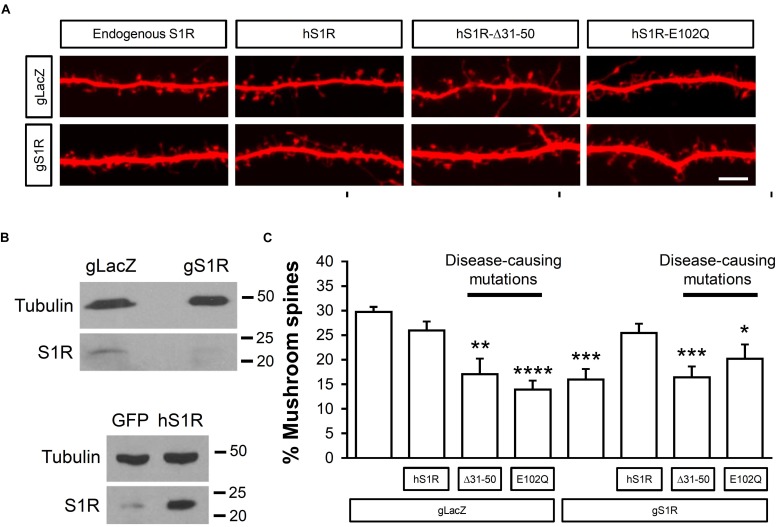
ALS and dHMN-linked mutations in S1R destabilize mushroom spines in hippocampal neurons. **(A)** Confocal images of dendrites and spines expressing TdTomato in WT hippocampal cultures that were infected with lenti-Cas9 and lenti-gLacZ or lenti-gS1R as well as human S1R (hS1R) with or without E102Q and Δ31–50 mutations in S1R on DIV7 and fixed on DIV19. Scale bar = 5 μm. **(B)** Western blotting confirmed deletion of S1R and overexpression of S1R. **(C)** Quantitative summary of mushrooms spine prevalence in WT hippocampal cultures (*N* = 5). ^∗^*p* < 0.05, ^∗∗^*p* < 0.01, ^∗∗∗^*p* < 0.001, ^∗∗∗∗^*p* < 0.0001.

Here we used CRISPR/Cas9 to delete endogenous S1R and evaluate functional effects of expression of hS1R with amyotrophic lateral sclerosis (ALS) (E102Q) and distal hereditary motor neuropathy (dHMN) (Δ31–50) causing mutations. For this, on DIV7 hippocampal cultures were transfected with TdTomato to visual spine morphology and infected with lentiviruses to encode Cas9 and sgRNA targeting either the *S1R* gene (gS1R) or the bacterial *lacZ* gene as a control (gLacZ). As previously reported (Tsai et al., 2009; Fisher et al., 2016; Ryskamp et al., 2019), lack of endogenous S1R led to a reduction in the prevalence of mushroom spines (Figures 1A,C). S1R deletion and lentivirus-mediated overexpression of hS1R were confirmed by Western blotting for hS1R (Figure 1B). Overexpression of hS1R-mutant constructs was confirmed in HEK293T cells (Supplementary Figure S1). Overexpression of hS1R was well-tolerated in gLacZ-treated cultures and it reinstated S1R’s basal role in supporting mushroom spine stability in gS1R-treated cultures (Figures 1A,C). Overexpression of E102Q or Δ31–50 mutants of hS1R destabilized mushroom spines in gLacZ-treated cultures and was unable to substitute for endogenous S1R (Figures 1A,C). This suggests that these mutations have a dominant-negative function and impair the basal activity of S1R that is important for neuronal function. Most likely overexpressed E102Q or Δ31–50 mutants replace endogenous S1R, resulting in loss of its activity and destabilization of mushroom synaptic spines. It is also possible that there is a gain of toxic function, but this is less likely as the mutations are recessive.

### *In silico* Docking of Pridopidine, (+)-3-PPP, and (+)-Pentazocine Predicts Important Residues for the Binding of Each S1R Agonist

**FIGURE 2:**
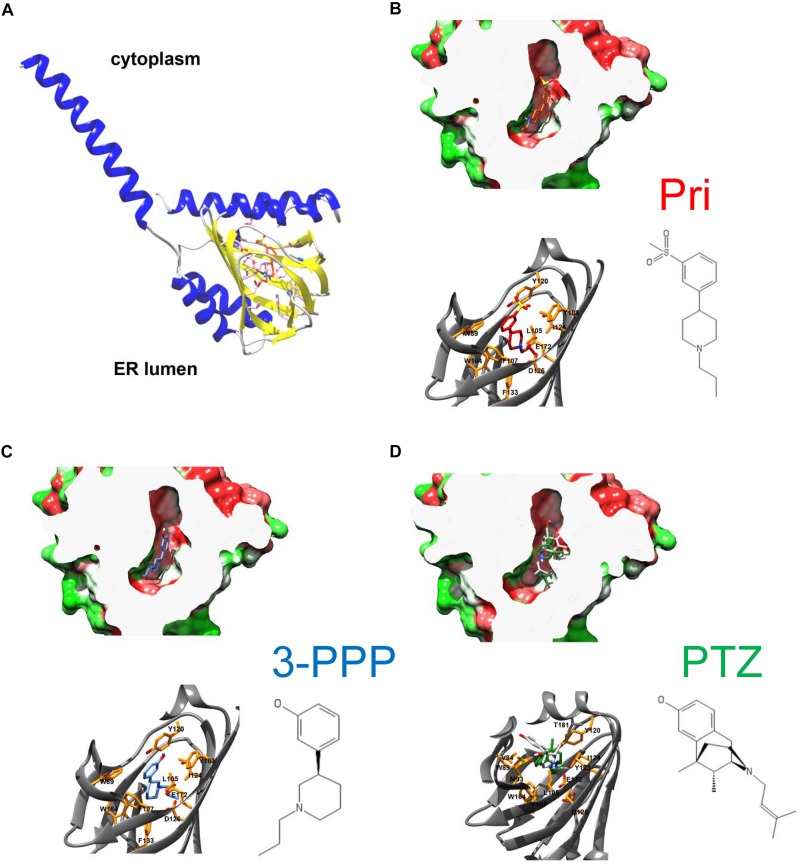
Overall architecture of S1R and docked structures of pridopidine, (+)-3-PPP, and (+)-PTZ. **(A)** The structure of human S1R based on Schmidt et al. (2016) with the orientation based on Mavylutov et al. (2018). S1R is a single transmembrane protein with the C-terminal ligand-binding domain residing in the lumen of endoplasmic reticulum. **(B–D)** Docked structures of pridopidine (**B**, ligand in red), (+)-3-PPP (**C**, ligand in blue), and (+)-PTZ (**D**, ligand in green) shown in a hydrophobicity-colored ligand-binding cavity (green-hydrophilic and red-hydrophobic). The position of PTZ according to the recently determined X-ray structure is shown in gray on panel **D**. Critical residues that form ligand-binding cavity are shown on each panel.

To gain insight into residues that are important for pridopidine and (+)-3-PPP binding, we performed *in silico* ligand docking studies using the hS1R crystal structure obtained by Schmidt et al. (2016) (PDB IDs 5HK1, 5HK2). According to the lipid cubic phase-crystal structure, S1R is a single-transmembrane receptor (Schmidt et al., 2016) and APEX2 fusion experiments suggest that its C-terminal ligand binding domain faces ER luminal side (Mavlyutov et al., 2017; Mavylutov et al., 2018; Figure 2A). Recently, the hS1R structure was also solved in complex with the classical S1R agonist (+)-PTZ (Schmidt et al., 2018). As a dextrorotatory benzomorphan, (+)-PTZ harbors a phenylpiperidine moiety similar to (+)-3-PPP and pridopidine, but it is more conformationally constrained. Nevertheless, the structural similarities between (+)-PTZ and (+)-3-PPP and pridopidine as well as pharmacological data indicate that they are all S1R agonists (Sahlholm et al., 2013, 2015; Ryskamp et al., 2017, 2019) and suggested that we could use the published S1R crystal structures to model (+)-3-PPP and pridopidine binding and examine important interacting residues. We modeled pridopidine, (+)-3-PPP, and PTZ docking to the ligand binding site and display residues that are predicted to directly interact with these S1R agonists (Figures 2B–D). *In silico* docking with (+)-PTZ was more difficult than with pridopidine or (+)-3-PPP because it is more bulky and is more conformationally restricted. The docked PTZ structure (Figure 2D, green) was similar to that determined by Schmidt et al. (2018) (Figure 2D, gray), but not completely identical, possibly due to the rigid receptor docking algorithm used in this study. Also, 3-PPP and pridopidine are more similar to the ligands that were used to generate the original S1R crystal structure.

**FIGURE 3:**
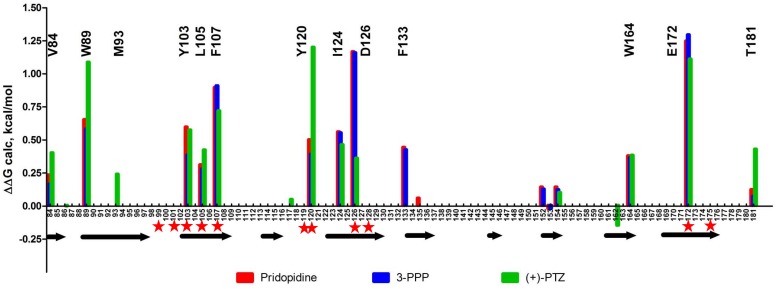
*In silico* alanine mutagenesis of S1R structures bound to pridopidine, (+)-3-PPP, and (+)-PTZ ligands. Bars indicate energetic contributions of individual residues involved in the formation of ligand binding pocket (red for pridopidine, blue for 3-PPP, and green for PTZ). Critical residues are marked on top of the graph. Secondary structure assignment is given below with each arrow corresponding to a beta-barrel forming strand. Previously reported critical ligand-binding residues are marked with red asterisks.

We also examined the predicted energetic contributions of each individual residue to pridopidine, (+)-3-PPP, and PTZ binding through *in silico* alanine mutagenesis (Figure 3). The ligand binding site of S1R is quite hydrophobic, with the exception of charged amino acids D126 and E172, which form a hydrogen bond with each other (Schmidt et al., 2016). They were identified as the most important residues for ligand binding because they interact with the positively charged nitrogen atom of these S1R agonists, a common structural feature of sigma-ligands. Additionally, Y103 creates a hydrogen bond with E172, contributing to formation of the binding pocket (Yamamoto et al., 1999). The polar SO_2_ group of pridopidine faces the beta-barrel opening, located close to the membrane surface. Y103 forms a hydrogen bond with the polar SO_2_ group. Aside from participating in hydrogen bonding, sulfonyl groups are relatively inert and are non-oxidizing. All other interactions mostly involve bulky aromatic residues forming numerous hydrophobic Van der Waals interactions, and there is very little solvent accessible area, as the ligands fit tightly in the binding pocket.

Overall, the PTZ-docked structure is similar to pridopidine/3-PPP structures with its polar group oriented toward the beta-barrel opening. Negatively charged groups of D126 and E172 also interact with the charged nitrogen moiety of PTZ. In addition, Y120 plays a role in hydrogen bonding to nitrogen as well as Van der Waals interactions. The polar hydroxyl group forms an H-bond with M93. There is a solvent accessible area near pocket opening which can be explained in part by our docking approach that did not allow movement of receptor side chains. Once again, E172 and D126 are predicted to be critical residues, together with Y120, Y103, W89, F107, and several other residues.

### Mutating S1R’s Drug-Binding Site Disrupts Rescue of Mushroom Spines by Pridopidine in a Model of Familial AD

**FIGURE 4:**
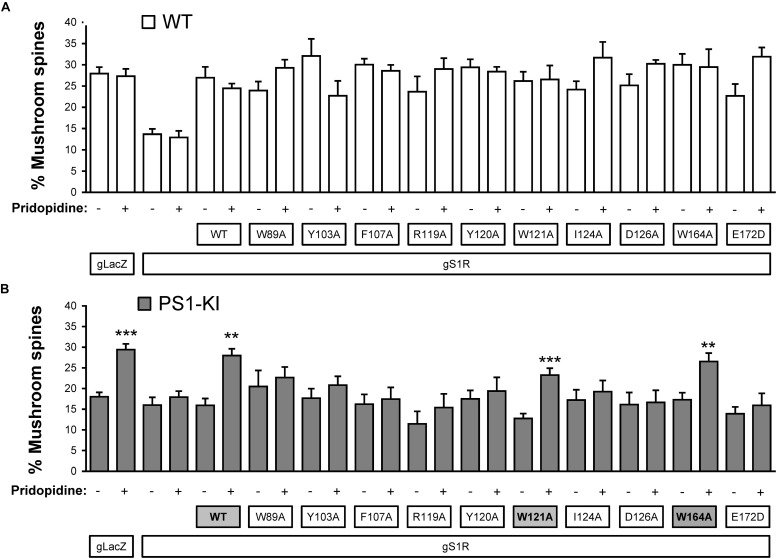
Mushroom spine rescue with S1R ligand binding site mutants. **(A,B)** Quantitative summary of mushrooms spine prevalence in WT **(A)** and PS1-KI **(B)** hippocampal cultures infected with lenti-Cas9 and lenti-lacZ (control) or lenti-gS1R (S1R KO). Human S1R (WT) or S1R ligand binding site mutant constructs were overexpressed by lentiviral infection on DIV7 as indicated. These cultures were transfected on DIV7 with a TdTomato plasmid using a high calcium phosphate method. At DIV18 cultures were treated for 16 h with the vehicle or 1 μM pridopidine, were fixed and TdTomato-expressing neurons were imaged with a confocal microscope. *N* = 3–16/condition in WT cultures and *N* = 5–16/condition in PSK-KI cultures. S1R constructs that retained the ability to rescue PS1-KI mushroom spines upon pridopidine treatment are shaded on panel **B** for clarity. ^∗∗^*p* < 0.01, ^∗∗∗^*p* < 0.001.

Based on our *in silico* studies (Figure 3) as well as previously reported effects of S1R mutations on PTZ-binding efficiency (Ossa et al., 2017), we generated several hS1R constructs with point mutations predicted to compromise S1R ligand binding (Figure 3). Expression of these S1R mutants was confirmed in experiments with HEK293T cells (Supplementary Figure S1). Binding of phenylpiperidine ligands was predicted to be impaired by W89A, Y103A, F107A, R119A, I124A, D126A, and E172D; modestly impaired by L105W, Y120A, and W164A; and not affected by W121A and I178A. R119 was not identified as an important residue in this study, but this mutation was previously reported to affect ligand binding (Ossa et al., 2017). We replaced L105 with a tryptophan side chain to shrink the ligand-binding site and restrict ligand entry. We tested these constructs in the absence of endogenous S1R, which resulted in mushroom spine loss as before. Interestingly, all studied mutations did not impair the basal function of S1R in WT mushroom spine stability, as they were able to bolster mushroom spines in the absence of endogenous S1R (Figure 4A). We previously reported a mushroom spine loss phenotype in hippocampal cultures prepared from PS1-KI mice, which model familial AD (Sun et al., 2014). This phenotype was recapitulated *in vivo* in aged PS1-KI mice (Sun et al., 2014) and was also observed in APP knock-in mice (Zhang et al., 2015). We recently found that pridopidine treatment stabilizes mushroom spines in these models of AD, but not in the absence of endogenous S1R, indicating that observed beneficial effects are mediated through ligand-dependent activation of S1R (Ryskamp et al., 2019). We replicated our previous findings (Ryskamp et al., 2019) that pridopidine treatment (1 μM for 16 h starting on DIV18) increases the prevalence of mushroom spines in PS1-KI cultures, but not in the absence of endogenous S1R (Figure 4B). Likewise, pridopidine is not able to compensate for mushroom spine loss caused by S1R deletion in WT cultures (Figure 4A). Although overexpression of hS1R can substitute for endogenous S1R in WT cultures (Figure 4A), it was insufficient to stabilize mushroom spines without a pharmacological boost from pridopidine (Figure 4B).

Consistent with the *in silico* predictions (Figure 3) and previous reports (Ossa et al., 2017), most drug-binding site mutations prevented the rescue of PS1-KI mushroom spines by pridopidine (W89A, Y103A, L105W, F107A, R119A, Y120A, I124A, D126A, E172A), whereas mutations that were not critical for drug binding did not impair the rescue of PS1-KI mushroom spines by pridopidine (W121A, W164A, I178A) (Figure 4B). These results indicate that a direct interaction between S1R and pridopidine underlies the synaptoprotective properties of pridopidine. They also indicate that while basal S1R activity is required for WT mushroom spine stability in hippocampal neurons, but it is insufficient to overcome the synaptic pathology associated with AD-causing mutations without agonist stimulation. This implies that basal S1R activity and agonist-stimulated S1R activity may employ different mechanisms to support mushroom spine stability.

## Discussion

### S1R Mutations and Neurodegenerative Disease

Several autosomal recessive mutations in S1R are linked to ALS (Luty et al., 2010; Al-Saif et al., 2011; Kim et al., 2014; Li et al., 2015; Ullah et al., 2015; Gregianin et al., 2016; Horga et al., 2016; Watanabe et al., 2016; Wong et al., 2016), but how these mutations impact S1R’s role in synaptic stability has not been previously investigated. Splicing site mutation in S1R gene caused in frame deletion of 20 amino acids (Δ31–50), leading to dHMN (Li et al., 2015). It was proposed that the ALS-linked mutation E102Q leads to protein aggregation, induction of the unfolded protein response pathway, defective autophagosome degradation, and impairments in vesicular transport (Dreser et al., 2017). Several reports indicate that the loss of S1R can exacerbate pathology and progression of SOD1^G93A^-linked ALS and other neurological disorders (Mavlyutov et al., 2011, 2013; Ha et al., 2012; Francardo et al., 2014; Miki et al., 2015; Maurice et al., 2018). Additionally, S1R dysfunction may contribute to AD pathology. S1R polymorphisms Q2P and C240T/G241T appear to modify AD risk in carriers of the APOE ε4 allele (Uchida et al., 2005; Maruszak et al., 2007; Huang et al., 2011; Fehér et al., 2012). The C240T/G241T allele is associated with downregulation of S1R (Mishina et al., 2008), which is significant because reduced expression of S1R may contribute to AD pathology. Positron emission tomography (PET) imaging shows that the binding of radiolabeled S1R ligands is reduced in several brain regions early in AD (Mishina et al., 2008). S1R-binding sites are also reduced in the CA1 region of the hippocampus in cadavers and this was correlated to loss of pyramidal cells (Jansen et al., 1993). This suggests that S1R may have a role in supporting the long-term viability of hippocampal neurons. Indeed, knockdown of S1R in hippocampal neurons leads to a loss of mushroom spines (Tsai et al., 2009; Fisher et al., 2016; Ryskamp et al., 2019) and S1R inhibits tau hyperphosphorylation by enhancing degradation of p35, an activator cyclin-dependent kinase 5 (Tsai et al., 2015). Thus, downregulation of S1R protein levels could contribute to pathology in AD and these observations could explain how loss of function mutations in S1R contribute to memory impairment in frontotemporal dementia (FTD).

We found that overexpression of hS1R is well-tolerated by WT hippocampal neurons, but it does not lead to an increase in dendritic spine density (not shown) or the percentage of mushroom spines. However, overexpression of S1R with ALS and dHMN-linked mutations (E102Q and Δ31–50) destabilized mushroom spines in WT cultures (Figure 1). Deletion of S1R (by Cas9 with gRNA targeting S1R) resulted in mushroom spine loss (Figure 1). Expression of hS1R prevented loss of mushroom spines in S1R knockout neurons, but this rescue was ineffective when hS1R had the E102Q or Δ31–50 mutations (Figure 1).

Amyotrophic lateral sclerosis and dHMN-causing mutations in S1R are recessive (Luty et al., 2010; Al-Saif et al., 2011; Kim et al., 2014; Li et al., 2015; Ullah et al., 2015; Gregianin et al., 2016; Horga et al., 2016; Watanabe et al., 2016), and we were surprised to see mushroom spine loss from overexpression of hS1R-Δ31–50 or hS1R-E102Q in hippocampal cultures that still had endogenous S1R. This is likely to be due to high levels of expression of S1R mutants in these experiments, leading to displacement of endogenous S1R. These results also potentially raise the possibility of subtle functional impairment in heterozygous carriers of these mutations. As neurons in culture are likely to be stressed compared to neurons *in vivo*, it is possible that synaptic deficits related to S1R inactivity only manifest *in vivo* when neurons are challenged by injury, inflammation, oxidative damage, aging, and/or other stressors.

### Structural Analysis of Drug-Binding Mutations in S1R

Our previous results suggested that pridopidine likely supports mushroom spines *in vitro* and *in vivo* in AD models by acting as an S1R agonist (Ryskamp et al., 2019). In the present study, we utilized mushroom spine rescue assay to analyze critical residues in S1R involved in functional effects of pridopidine. To accomplish this, we deleted endogenous S1R with the CRISPR/Cas9 system in WT and PS1-KI cultures and replaced it with the human version of S1R with or without mutations predicted to disrupt drug binding. These mutations were selected based on our *in silico* analysis (Figures 2, 3) and PTZ-binding data with various S1R mutations summarized in Ossa et al. (2017). We summarized our findings and previously reported drug-binding mutation data in Table 1. hS1R with or without ligand-binding mutations compensated for endogenous S1R in maintaining WT mushroom spine stability (Figure 4A). hS1R, hS1R-W121A, hS1R-W164A, hS1R-I178A overexpression, but not hS1R-W89A, hS1R-Y103A, hS1R-L105W, hS1R-F107A, hS1R-R119A, hS1R-Y120A, hS1R-I124A, hS1R-D126A, or hS1R-E172D overexpression, enabled the pridopidine-dependent rescue of PS1-KI mushroom spines in the absence of endogenous S1R (Figure 4B). Thus, the interaction between S1R and pridopidine is required for the rescue of PS1-KI mushroom spines in hippocampal cultures. These data are consistent with our *in silico* modeling (Figures 2, 3) and prior PTZ-binding data (Ossa et al., 2017), supporting the validity of the hS1R crystal structure model of S1R topology (Table 1). We are not able to predict effects of L105W as we used alanine scanning method in our *in silico* modeling (Figure 3). The main difference between *in silico* predictions and spine rescue data is related to the R119A mutation (Table 1). R119 residue is solvent exposed in the structure and facing outside of the binding pocket (Figure 2), but the Ala mutation in this residue abolished the effects of pridopidine (Figure 4B) and also resulted in loss of ligand binding (Ossa et al., 2017; Table 1). We cannot rule out that R119A mutation altered the overall secondary structure of S1R and affected ligand binding site of S1R indirectly or affected association of S1R with downstream effectors. Although *in silico* modeling has helped us to confirm the importance of several residues that are critical for the effects of pridopidine, studies involving co-crystalization of S1R and pridopidine as well as drug-binding studies with S1R point mutants would further refine our understanding of how this drug acts upon S1R to confer synaptoprotection.

**TABLE 1:** Analysis of S1R mutants in residues potentially involved in ligand binding.

**S1R residue/mutation tested**	**Importance of original residue for ligand binding [data summarized by Ossa et al. (2017)]**	**Predicted importance of residue for S1R ligand docking in crystal structure**	**Importance of residue for rescue of PS1-KI mushroom spines by pridopidine**
W89A	N/A	+++	+++
Y103A	+++	+++	+++
L105W	+	N/A	+++
F107A	+++	+++	+++
R119A	+++	−	+++
Y120A	+	+++	+++
W121A	−	−	−
I124A	N/A	+++	+++
D126A	+++	+++	+++
D164A	N/A	+	−
E172D	+++	+++	+++
I178A	N/A	−	−

*+++, important; +, moderately important; −, not important; N/A, data not available.*

Sigma-1 receptor exists in monomeric and multiple homooligomeric forms and the shifting balance between these states by agonists and antagonists may have relevance for S1R activity (Gromek et al., 2014; Mishra et al., 2015; Hong et al., 2017). However, the functional importance of this for synaptic biology has not been explored. Oligomerization of S1R protomers requires a GXXXG motif (corresponding to amino acid residues 87–91 of S1R) (Aydar et al., 2002; Gromek et al., 2014; Ortega-Roldan et al., 2015). There is conflicting data regarding the effect of S1R ligands on S1R oligomerization. Gromek et al. (2014) found that ligand binding favors the oligomer state of recombinant protein *in vitro*. By contrast, Mishra et al. (2015) and Hong et al. (2017) observed that agonists favor subunit dissociation using spectroscopic FRET and BRET approaches. It is currently unknown whether S1R monomers and/or oligomers contribute to mushroom spine stability and how their functions are modified by pridopidine. Future experiments are needed to test whether the oligomeric or homomeric form of S1R can stabilize PS1-KI mushroom spines in response to agonist stimulation.

### S1R May Support Mushroom Spine Stability Through Multiple Mechanisms

Knockdown of S1R causes mushroom spine loss in WT hippocampal cultures (Tsai et al., 2009; Fisher et al., 2016) and this cannot be rescued by pridopidine (Ryskamp et al., 2019). Overexpression of hS1R can substitute for endogenous S1R and stabilize WT mushroom spines (Figures 1, 4; Ryskamp et al., 2019); however, this is insufficient to restore mushroom spines to WT levels in PS1-KI hippocampal cultures (Figure 4B). Pridopidine increases the prevalence of PS1-KI mushroom spines to WT levels, but not in the absence of endogenous S1R (Figure 4B). Thus, basal S1R activity is needed for stability of WT mushroom spines, but agonist-mediated activation of S1R is necessary to prevent spine loss in PS1-KI neurons. Likewise, S1R knockdown causes spine loss in MSNs and pridopidine can prevent spine loss in YAC128 MSNs, but not in the absence of S1R (Ryskamp et al., 2017). The basis for distinct roles of S1R in mushroom spine stability in health and disease is only beginning to be understood. S1R knockout data indicate that basal S1R activity may be important for mitigating oxidative stress and regulating the actin cytoskeleton via Rac-GTP signaling (Tsai et al., 2009), whereas the agonist-mediated rescue of HD MSN spines or AD mushroom spines may involve normalization of calcium signaling (Wu et al., 2016, 2018; Ryskamp et al., 2017, 2019). Although S1R immunostaining has been detected in postsynaptic spines using a custom antibody and electron microscopy (Alonso et al., 2000), it remains unknown whether S1R stabilizes spines by acting at synapses locally or if it exerts more global neuroprotective effects.

## Conclusion

Sigma-1 receptor agonists (e.g., pridopidine) can normalize synaptic connectivity in mouse models of AD. We prepared hippocampal cultures from WT mouse pups and deleted S1R with Cas9. In the absence of S1R, cultured hippocampal neurons had fewer mushroom spines. Overexpression of hS1R restored spines to gLacZ levels. By contrast, overexpression of S1R with the Δ31–50 deletion (linked to dHMN) or the E102Q mutation (linked to ALS) destabilized spines in WT cultures. We previously found that pridopidine requires S1R for its beneficial effects in cellular models of AD. We report here that the synaptoprotective effects of pridopidine require a direct interaction with S1R, as drug-binding site mutations disrupted the spine rescue in a culture model of familial AD. Our mutational analysis was generally in agreement with *in silico* docking based on the published S1R crystal structure, with an exception of the R119 residue. Our data also suggest that basal S1R activity is required for mushroom spine stability, whereas agonist-mediated S1R activity is required for stabilization of mushroom spines in the context of disease-causing mutations. Obtained results provide novel insights regarding S1R function in the nervous system.

## Data Availability

The datasets generated for this study are available on request to the corresponding author.

## Ethics Statement

The animal study was reviewed and approved by the Institutional Animal Care and Use Committee of the University of Texas Southwestern Medical Center at Dallas and followed the National Institutes of Health Guidelines for the Care and Use of Experimental Animals.

## Author Contributions

DR performed the experiments with neuronal cultures. VZ performed the docking and modeling studies. IB initiated and supervised the research, and prepared the final version of the manuscript for publication.

## Conflict of Interest Statement

The authors declare that the research was conducted in the absence of any commercial or financial relationships that could be construed as a potential conflict of interest.

## Footnotes

1 addgene.org/14883/

2 addgene.org/52963/

3 addgene.org/52962/

## Supplementary Material

FIGURE S1 Western blotting confirms overexpression of human S1R (hS1R) and S1R-mutant constructs in HEK293T cells. HEK293T cells were transiently transfected with plasmids encoding GFP, hS1R, or hS1R with the indicated mutations. Cells were lysed 48 h later and protein was extracted for analysis by Western blotting.
