# Supplementary figures and images for: Mutational Analysis of Sigma-1 Receptor’s Role in Synaptic Stability
Source: Front Neurosci. 2019 Sep 19;13:1012. doi: 10.3389/fnins.2019.01012 (PMC6761230; doi:10.3389/fnins.2019.01012)

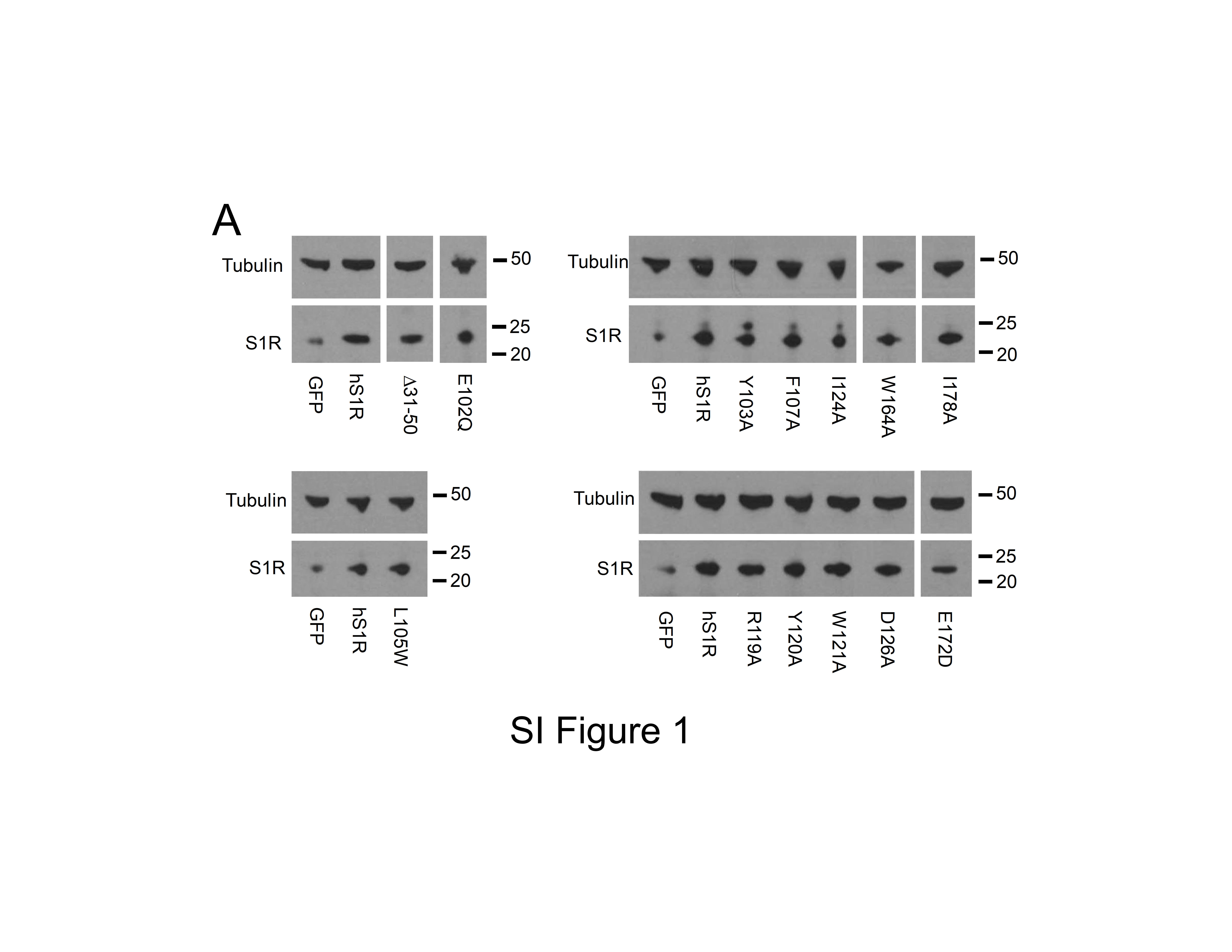

Supplement: FIGURE S1 — Western blotting confirms overexpression of human S1R (hS1R) and S1R-mutant constructs in HEK293T cells. HEK293T cells were transiently transfected with plasmids encoding GFP, hS1R, or hS1R with the indicated mutations. Cells were lysed 48 h later and protein was extracted for analysis by Western blotting. [file Image_1.JPEG]
